# Strong convergence and bounded perturbation resilience of a modified proximal gradient algorithm

**DOI:** 10.1186/s13660-018-1695-x

**Published:** 2018-05-02

**Authors:** Yanni Guo, Wei Cui

**Affiliations:** College of Science, Civil Aviation University of China, Tianjin, China

**Keywords:** Strong convergence, Bounded perturbation resilience, Modified proximal gradient algorithm, Viscosity approximation, Convex minimization problem

## Abstract

The proximal gradient algorithm is an appealing approach in finding solutions of non-smooth composite optimization problems, which may only has weak convergence in the infinite-dimensional setting. In this paper, we introduce a modified proximal gradient algorithm with outer perturbations in Hilbert space and prove that the algorithm converges strongly to a solution of the composite optimization problem. We also discuss the bounded perturbation resilience of the basic algorithm of this iterative scheme and illustrate it with an application.

## Introduction

Let *H* be a real Hilbert space with an inner product $\langle\cdot ,\cdot\rangle$ and an induced norm $\Vert\cdot\Vert$. Let $\Gamma_{0}(H)$ be the class of convex, lower semi-continuous, and proper functions from *H* to $(-\infty, +\infty]$. Consider the following non-smooth composite optimization problem:

1 $$ \min_{x\in H}\bigl(f(x)+g(x)\bigr), $$

where $f, g\in\Gamma_{0}(H)$, *f* is differentiable and ∇*f* is *L*-Lipschitz continuous on *H* with $L>0$. *g* may not be differentiable. If further, $f+g=:\Phi$ is coercive, that is,

2 $$ \lim_{ \Vert x \Vert \rightarrow+\infty}\Phi(x)=+\infty, $$

then Φ has a minimizer over *H*, that is, $S:=\operatorname{Argmin}(\Phi)\neq \emptyset$, see [1, page 159, Proposition 11.14]. Problem (1) has a typical scenario in linear inverse problems [2], it has applications in compressed sensing, machine learning, data recovering and so on (see [3–6] and the references therein).

Proximal gradient methods are among the methods used for solving problem (1), which allow to decouple the contribution of the functions *f* and *g* in a gradient descent step determined by *f* and in a proximal step induced by *g* [7, 8]. For the classical proximal gradient method, the initial value $x_{0}\in H$ is given, and the iterative algorithm for generating sequence $\{x_{n}\}$ is defined as follows:

3 $$ x_{n+1}=\operatorname{prox}_{\lambda g}(I-\lambda \nabla f) (x_{n}), \quad \forall n\geq0, $$

where $\lambda>0$ is the step size, $\operatorname{prox}_{\lambda g}$ is a proximal operator (see Sect. 2). If $S\neq\emptyset$ and $0<\lambda<\frac{2}{L}$, then any sequence generated by algorithm (3) converges weakly to an element of *S* [1, Corollary 27.9]. Xu [9] put forward the following slightly more general proximal gradient algorithm:

4 $$ x_{n+1}=\operatorname{prox}_{\lambda_{n}g}(I- \lambda_{n}\nabla f) (x_{n}) $$

for problem (1), where the weak convergence of the generated sequence $\{x_{n}\}$ was obtained. Besides, it was noted that no strong convergence is guaranteed if $\dim H=\infty$. In 2017, Guo, Cui and Guo [10] proposed the following proximal gradient algorithm with perturbations:

5 $$ x_{n+1}=\operatorname{prox}_{\lambda_{n}g}(I- \lambda_{n}D\nabla f+e) (x_{n}). $$

The generated sequence $\{x_{n}\}$ again converges weakly to a solution of (1).

On the other hand, it is well known that the viscosity approximation method proposed by Moudafi [11] generates a sequence $\{x_{n}\}$:

6 $$ x_{n+1}=t_{n}h(x_{n})+(1-t_{n})Tx_{n}, $$

which converges strongly to a fixed point $x^{*}$ of *T* for some contractive operator *h*. In 2004, Xu [12, Theorem 3.1] further proved that the above $x^{*}$ is also the unique solution of the variational inequality:

7 $$ \bigl\langle (I-h)x^{*},x-x^{*}\bigr\rangle \geq0,\quad\forall x\in \operatorname{Fix}(T), $$

provided that $\{t_{n}\}$ satisfies certain conditions.

This paper is based on viscosity algorithm (6) and proximal gradient algorithm (4) to generate a sequence with perturbations, which converges strongly to a solution of problem (1). We also apply this algorithm to solve the linear inverse problem.

An objective of this paper considering the perturbation is the superiorization methodology introduced by [13]. The superiorization method may not find an optimal solution to the given objective function. It might try to find a point with a lower cost function value than other points by a rather simple algorithm, which is known as the basic algorithm (see [14–18] for more details). It is a heuristic method with less time consuming that makes it applicable to some important practical problems such as medical image recovery [19, 20], computed tomography [21], intensity-modulated radiation therapy [22] and the like. However, the superiorization method needs to investigate the basic iterative algorithm’s bounded perturbation resilience. Hence, there raises a new problem whether the basic algorithm is bounded perturbation resilient. Very recently, several articles focused on this topic [23–27]. So another task of this paper is to discuss the bounded perturbation resilience of the modified proximal gradient algorithm.

### Results and discussion

In view of the facts that the sequence generated by (5) converges weakly to a solution of (1), the viscosity method can convert a weakly convergent sequence to a strongly convergent one, and that the applied widely superiorization method introduced by [13] is based on the bounded perturbation resilience of basic algorithms, we discuss the strong convergence problem of a modified proximal gradient algorithm with perturbations as well as the bounded perturbation resilience of the responding basic algorithm.

The structure of this paper is as follows. In Sect. 2, we introduce some definitions and lemmas that will be used to prove the main results in the subsequent sections. In Sect. 3, we present the modified proximal gradient algorithm with perturbations and prove that the generated sequence $\{x_{n}\}$ converges strongly to a solution of problem (1). We conclude this section with several corollaries. In Sect. 4, we introduce the definition of bounded perturbation resilience and certify the corresponding strong convergence result. In Sect. 5, we apply our algorithm to the linear inverse problem, and illustrate it with a specific numerical example. Finally, we give a conclusion in Sect. 6.

## Preliminaries

Let $\{x_{n}\}$ be a sequence in Hilbert space *H* and $x\in H$. Let $T:H\rightarrow H$ be an operator (linear or nonlinear). We list some notations.

$x_{n}\rightarrow x$ means $\{x_{n}\}$ converges strongly to *x*.

$x_{n}\rightharpoonup x$ means $\{x_{n}\}$ converges weakly to *x*.

If there exists a subsequence $\{x_{n_{j}}\}$, which converges weakly to a point *z*, we will call *z* a weak cluster point of $\{x_{n}\}$. The set of all cluster points of $\{x_{n}\}$ is denoted by $\omega_{w}(x_{n})$.

$\operatorname{Fix}(T):=\{x\in H: Tx=x\}$.

The following definitions are needed in proving our main results.

### Definition 2.1

Let *T*, $A:H\rightarrow H$ be operators.

(i) *T* is nonexpansive if
  $$ \Vert Tx-Ty \Vert \leq \Vert x-y \Vert , \quad \forall x,y\in H. $$
(ii) *T* is *L*-Lipschitz continuous with $L\geq0$, if
  $$ \Vert Tx-Ty \Vert \leq L \Vert x-y \Vert , \quad \forall x,y\in H. $$
  We call *T* a contractive mapping if $0\leq L<1$.
(iii) *T* is *α*-averaged if
  $$ T=(1-\alpha)I+\alpha S, $$
  where $\alpha\in(0,1)$, and $S:H\rightarrow H$ is nonexpansive.
(iv) *A* is *v*-inverse strongly monotone (*v*-ism) with $v>0$, if
  $$ \langle Ax-Ay, x-y\rangle\geq v \Vert Ax-Ay \Vert ^{2}, \quad \forall x,y\in H. $$

Given $g\in\Gamma_{0}(H)$, [1, Proposition 12.15] ensures that $\frac{ \Vert y-x \Vert^{2}}{2}+g(y)$ has exact one minimizer over *H* for each $x\in H$. So we have

### Definition 2.2

(Proximal operator)

Let $g\in\Gamma_{0}(H)$. The proximal operator of *g* is defined by

8 $$ \operatorname{prox}_{g}(x):=\arg\min _{y\in H}\biggl\{ \frac{ \Vert y-x \Vert ^{2}}{2}+g(y)\biggr\} , \quad x\in H. $$

The proximal operator of *g* of order $\alpha>0$ is defined as the proximal operator of *αg*. Moreover, it satisfies (see [3, Remark 12.24])

9 $$ \operatorname{prox}_{\alpha g}(x):=\arg\min _{y\in H}\biggl\{ \frac{ \Vert y-x \Vert ^{2}}{2\alpha}+g(y)\biggr\} , \quad x\in H. $$

The following lemmas (Lemma 2.3 and Lemma 2.4) describe the properties of the proximal operators.

### Lemma 2.3

([9, Lemma 3.1])

*Let* $g\in\Gamma_{0}(H)$, *and* $\alpha>0$, $\mu>0$. *Then*

$$\operatorname{prox}_{\alpha g}(x)=\operatorname{prox}_{\mu g}\biggl( \frac{\mu }{\alpha}x+\biggl(1-\frac{\mu}{\alpha}\biggr)\operatorname{prox}_{\alpha g}x \biggr). $$

### Lemma 2.4

([8, Lemma 2.4], [1, Remark 4.24])

*Let* $g\in\Gamma_{0}(H)$, *and* $\alpha>0$. *Then the proximity operator* $\operatorname{prox}_{\alpha g}$ *is* $\frac{1}{2}$-*averaged*. *In particular*, *it is nonexpansive*, *that is*,

10 $$ \bigl\Vert \operatorname{prox}_{\alpha g}(x)-\operatorname {prox}_{\alpha g}(y) \bigr\Vert \leq \Vert x-y \Vert , \quad \forall x,y\in H. $$

### Lemma 2.5

([9, Proposition 3.2])

*Let* $f,g\in\Gamma_{0}(H)$, $z\in H$ *and* $\alpha>0$. *Assume that* *f* *is differentiable on* *H*. *Then* *z* *is a solution to* (1) *if and only if* *z* *solves the fixed point equation*

11 $$ z=\operatorname{prox}_{\alpha g}(I-\alpha\nabla f)z. $$

The following two lemmas play an important role in proving the strong convergence result.

### Lemma 2.6

([1, Theorem 4.17])

*Let* $T:H\rightarrow H$ *be a nonexpansive mapping with* $\operatorname{Fix}(T)\neq\emptyset$. *If* $\{x_{n}\}$ *is a sequence in* *H* *converging weakly to* *x*, *and if* $\{(I-T)x_{n}\}$ *converges strongly to* *y*, *then* $(I-T)x=y$.

### Lemma 2.7

([28, Lemma 2.5])

*Assume that* $\{a_{n}\}$ *is a sequence of nonnegative real numbers satisfying*

$$ a_{n+1}\leq(1-\gamma_{n})a_{n}+ \gamma_{n}\delta_{n}+\beta_{n}, \quad n\geq0, $$

*where* $\{\gamma_{n}\}$, $\{\delta_{n}\}$ *and* $\{\beta_{n}\}$ *satisfy the conditions*:

(i) $\{\gamma_{n}\}\subset[0,1]$, $\sum_{n=0}^{\infty}\gamma _{n}=\infty$, *or equivalently*, $\prod_{n=0}^{\infty}(1-\gamma_{n})=0$;
(ii) $\lim\sup_{n\rightarrow\infty}\delta_{n}\leq0$;
(iii) $\beta_{n}\geq0$ ($n\geq0$), $\sum_{n=0}^{\infty}\beta _{n}<\infty$.

*Then* $\lim_{n\rightarrow\infty}a_{n}=0$.

## Convergence analysis

In this section, let *H* be a Hilbert space, $h:H\rightarrow H$ a *ρ*-contractive operator with $\rho\in(0,1)$. $f,g\in\Gamma_{0}(H)$. *f* is differentiable, and ∇*f* is Lipschitz continuous with Lipschitz constant $L>0$. Given $x_{0}\in H$, we propose the following modified proximal gradient algorithm for solving (1):

12 $$ x_{n+1}:=t_{n}h(x_{n})+(1-t_{n}) \operatorname{prox}_{\alpha _{n}g}(I-\alpha_{n}\nabla f) (x_{n})+e(x_{n}), \quad n\geq0, $$

where $\{t_{n}\}$ is a sequence in $[0,1]$. $0< a=\inf_{n}\alpha_{n}\leq\alpha _{n}<\frac{2}{L}$. $e:H\rightarrow H$ represents a perturbation operator and satisfies

13 $$ \sum_{n=0}^{\infty} \bigl\Vert e(x_{n}) \bigr\Vert < +\infty. $$

We also introduce the following iterative scheme as a special case to (12):

14 $$ x_{n+1}:=t_{n}h(x_{n})+(1-t_{n}) \operatorname{prox}_{\alpha _{n}g}(I-\alpha_{n}\nabla f+e) (x_{n}), \quad n\geq0. $$

We state the main strong convergence theorem.

### Theorem 3.1

*Let* *S* *be the solution set of* (1), *and assume that* $S\neq \emptyset$. *Given* $x_{0}\in H$. *Let* $\{x_{n}\}$ *be generated by* (12). *If* (13) *and the following conditions hold*:

(i) $0< a=\inf_{n}\alpha_{n}\leq\alpha_{n}<\frac{2}{L}$, $\sum_{n=0}^{\infty}|\alpha_{n+1}-\alpha_{n}|<\infty$;
(ii) $\{t_{n}\}\subset(0,1)$, $\lim_{n\rightarrow\infty}t_{n}=0$;
(iii) $\sum_{n=0}^{\infty}t_{n}=\infty$, $\sum_{n=0}^{\infty }|t_{n+1}-t_{n}|<\infty$.

*Then* $\{x_{n}\}$ *converges strongly to a point* $x^{*}\in S$, *where* $x^{*}$ *is the unique solution of the following variational inequality problem*:

15 $$ \bigl\langle (I-h)x^{*},x-x^{*}\bigr\rangle \geq0,\quad\forall x\in S. $$

### Proof

We point out that $\operatorname{prox}_{\alpha_{n}g}(I-\alpha _{n}\nabla f)$ is nonexpansive for each *n*. Let us follow the proof of [29]. At first, that ∇*f* is *L*-Lipschizian means that ∇*f* is $\frac{1}{L}$-ism ([1, Theorem 18.15]). Consequently, $I-\alpha_{n}\nabla f$ is $\frac{\alpha_{n}L}{2}$-averaged as $0<\alpha _{n}<\frac{2}{L}$ ([1, Proposition 4.33]). Besides, $\operatorname{prox}_{\alpha_{n}g}$ is $\frac{1}{2}$-averaged by Lemma 2.4, the composite $\operatorname{prox}_{\alpha_{n}g}(I-\alpha_{n}\nabla f)$ is $\frac{\alpha_{n}L+2}{4}$-averaged ([29, Proposition 3.2]). Then it is nonexpansive ([1, Remark 4.24]).

Set $T_{n}=\operatorname{prox}_{\alpha_{n}g}(I-\alpha_{n}\nabla f)$. For any $\bar{x}\in S$, we have

$$\Vert T_{n}x_{n}-\bar{x} \Vert \leq \Vert x_{n}-\bar {x} \Vert $$

by applying Lemma 2.5 and that $T_{n}$ is nonexpansive. So,

16 $$\begin{aligned} & \Vert x_{n+1}-\bar{x} \Vert \\ &\quad = \bigl\Vert t_{n}h(x_{n})+(1-t_{n})T_{n}x_{n}+e(x_{n})- \bar{x} \bigr\Vert \\ &\quad \leq t_{n} \bigl\Vert h(x_{n})-\bar{x} \bigr\Vert +(1-t_{n}) \Vert T_{n}x_{n}-\bar{x} \Vert + \bigl\Vert e(x_{n}) \bigr\Vert \\ &\quad \leq t_{n} \bigl\Vert h(x_{n})-h(\bar{x}) \bigr\Vert +t_{n} \bigl\Vert h(\bar{x})-\bar{x} \bigr\Vert +(1-t_{n}) \Vert T_{n}x_{n}-\bar{x} \Vert + \bigl\Vert e(x_{n}) \bigr\Vert \\ &\quad \leq t_{n}\rho \Vert x_{n}-\bar{x} \Vert +t_{n} \bigl\Vert h(\bar{x})-\bar{x} \bigr\Vert +(1-t_{n}) \Vert x_{n}-\bar{x} \Vert + \bigl\Vert e(x_{n}) \bigr\Vert \\ &\quad =\bigl(1-t_{n}(1-\rho)\bigr) \Vert x_{n}-\bar{x} \Vert +t_{n}(1-\rho )\cdot\frac{ \Vert h(\bar{x})-\bar{x} \Vert }{1-\rho}+ \bigl\Vert e(x_{n}) \bigr\Vert \\ &\quad \leq\max \biggl\{ \Vert x_{n}-\bar{x} \Vert ,\frac{ \Vert h(\bar{x})-\bar{x} \Vert }{1-\rho} \biggr\} + \bigl\Vert e(x_{n}) \bigr\Vert . \end{aligned}$$

An induction argument shows that

$$ \Vert x_{n+1}-\bar{x} \Vert \leq\max \biggl\{ \Vert x_{0}-\bar{x} \Vert , \frac{ \Vert h(\bar{x})-\bar{x} \Vert }{1-\rho} \biggr\} +\sum _{k=0}^{\infty} \bigl\Vert e(x_{k}) \bigr\Vert . $$

Hence $\{x_{n}\}$ is bounded as $\sum_{n=0}^{\infty} \Vert e(x_{n}) \Vert <\infty$. Consequently, we get the boundedness of $\{h(x_{n})\}$ and $\{ T_{n}x_{n}\}$.

We next prove that $\Vert x_{n+1}-x_{n} \Vert\rightarrow0$ as $n\rightarrow\infty$. In fact,

17 $$\begin{aligned} & \Vert x_{n+1}-x_{n} \Vert \\ &\quad = \bigl\Vert t_{n}h(x_{n})+(1-t_{n})T_{n}x_{n}+e(x_{n})-t_{n-1}h(x_{n-1})-(1-t_{n-1})T_{n-1}x_{n-1} \\ &\qquad{}-e(x_{n-1}) \bigr\Vert \\ &\quad \leq \bigl\Vert t_{n}\bigl[h(x_{n})-h(x_{n-1}) \bigr]+\bigl[t_{n}h(x_{n-1})-t_{n-1}h(x_{n-1}) \bigr] \bigr\Vert \\ &\qquad{}+ \bigl\Vert (1-t_{n}) (T_{n}x_{n}-T_{n}x_{n-1})+(1-t_{n})T_{n}x_{n-1}-(1-t_{n-1})T_{n-1}x_{n-1} \bigr\Vert \\ &\qquad{}+ \bigl\Vert e(x_{n}) \bigr\Vert + \bigl\Vert e(x_{n-1}) \bigr\Vert \\ &\quad \leq t_{n}\rho \Vert x_{n}-x_{n-1} \Vert + \vert t_{n}-t_{n-1} \vert \bigl\Vert h(x_{n-1}) \bigr\Vert \\ &\qquad{}+(1-t_{n}) \Vert T_{n}x_{n}-T_{n}x_{n-1} \Vert +(1-t_{n}) \Vert T_{n}x_{n-1}-T_{n-1}x_{n-1} \Vert \\ &\qquad{}+ \vert t_{n}-t_{n-1} \vert \Vert T_{n-1}x_{n-1} \Vert + \bigl\Vert e(x_{n}) \bigr\Vert + \bigl\Vert e(x_{n-1}) \bigr\Vert \\ &\quad \leq t_{n}\rho \Vert x_{n}-x_{n-1} \Vert + \vert t_{n}-t_{n-1} \vert \bigl\Vert h(x_{n-1}) \bigr\Vert +(1-t_{n}) \Vert x_{n}-x_{n-1} \Vert \\ &\qquad{}+(1-t_{n}) \Vert T_{n}x_{n-1}-T_{n-1}x_{n-1} \Vert + \vert t_{n}-t_{n-1} \vert \Vert T_{n-1}x_{n-1} \Vert \\ &\qquad{}+ \bigl\Vert e(x_{n}) \bigr\Vert + \bigl\Vert e(x_{n-1}) \bigr\Vert \\ &\quad \leq\bigl(1-t_{n}(1-\rho)\bigr) \Vert x_{n}-x_{n-1} \Vert + \vert t_{n}-t_{n-1} \vert \bigl( \bigl\Vert h(x_{n-1}) \bigr\Vert + \Vert T_{n-1}x_{n-1} \Vert \bigr) \\ &\qquad{}+(1-t_{n}) \Vert T_{n}x_{n-1}-T_{n-1}x_{n-1} \Vert + \bigl\Vert e(x_{n}) \bigr\Vert + \bigl\Vert e(x_{n-1}) \bigr\Vert . \end{aligned}$$

By applying Lemma 2.3 and Lemma 2.4, we compute

18 $$\begin{aligned} & \Vert T_{n}x_{n-1}-T_{n-1}x_{n-1} \Vert \\ &\quad = \bigl\Vert \bigl(\operatorname{prox}_{\alpha_{n}g}(I-\alpha_{n} \nabla f)\bigr)x_{n-1}-\bigl(\operatorname{prox}_{\alpha_{n-1}g}(I- \alpha_{n-1}\nabla f)\bigr)x_{n-1} \bigr\Vert \\ &\quad = \biggl\Vert \bigl(\operatorname{prox}_{\alpha_{n}g}(I- \alpha_{n}\nabla f)\bigr)x_{n-1}-\operatorname{prox}_{\alpha_{n}g} \biggl(\frac{\alpha _{n}}{\alpha_{n-1}} (I-\alpha_{n-1}\nabla f)x_{n-1} \\ &\qquad{}+\biggl(1-\frac{\alpha_{n}}{\alpha_{n-1}}\biggr)\bigl[\operatorname {prox}_{\alpha_{n-1}g}(I-\alpha_{n-1}\nabla f)\bigr]x_{n-1} \biggr) \biggr\Vert \\ &\quad \leq \biggl\Vert (I-\alpha_{n}\nabla f)x_{n-1}- \frac{\alpha_{n}}{\alpha _{n-1}}(I-\alpha_{n-1}\nabla f)x_{n-1}- \biggl(1- \frac{\alpha_{n}}{\alpha _{n-1}} \biggr)T_{n-1}x_{n-1} \biggr\Vert \\ &\quad = \biggl\vert 1-\frac{\alpha_{n}}{\alpha_{n-1}} \biggr\vert \Vert x_{n-1}-T_{n-1}x_{n-1} \Vert \\ &\quad \leq\frac{ \vert \alpha_{n}-\alpha_{n-1} \vert }{a} \Vert x_{n-1}-T_{n-1}x_{n-1} \Vert , \end{aligned}$$

where $a=\inf_{n}\alpha_{n}>0$.

Substituting (18) into (17), we obtain

19 $$\begin{aligned} & \Vert x_{n+1}-x_{n} \Vert \\ &\quad \leq\bigl(1-t_{n}(1-\rho)\bigr) \Vert x_{n}-x_{n-1} \Vert + \vert t_{n}-t_{n-1} \vert \bigl( \bigl\Vert h(x_{n-1}) \bigr\Vert + \Vert T_{n-1}x_{n-1} \Vert \bigr) \\ &\qquad{}+(1-t_{n})\frac{ \vert \alpha_{n}-\alpha_{n-1} \vert }{a} \Vert x_{n-1}-T_{n-1}x_{n-1} \Vert + \bigl\Vert e(x_{n}) \bigr\Vert + \bigl\Vert e(x_{n-1}) \bigr\Vert \\ &\quad \leq\bigl(1-t_{n}(1-\rho)\bigr) \Vert x_{n}-x_{n-1} \Vert +\frac{ \vert \alpha_{n}-\alpha_{n-1} \vert }{a}\bigl( \Vert x_{n-1} \Vert + \Vert T_{n-1}x_{n-1} \Vert \bigr) \\ &\qquad{}+t_{n}(1-\rho)\cdot\frac{(-1)\cdot \vert \alpha_{n}-\alpha _{n-1} \vert }{a(1-\rho)} \bigl( \Vert x_{n-1} \Vert + \Vert T_{n-1}x_{n-1} \Vert \bigr) \\ &\qquad{}+ \vert t_{n}-t_{n-1} \vert \bigl( \bigl\Vert h(x_{n-1}) \bigr\Vert + \Vert T_{n-1}x_{n-1} \Vert \bigr)+ \bigl\Vert e(x_{n}) \bigr\Vert + \bigl\Vert e(x_{n-1}) \bigr\Vert \\ &\quad \leq\bigl(1-t_{n}(1-\rho)\bigr) \Vert x_{n}-x_{n-1} \Vert \\ &\qquad{}+t_{n}(1-\rho)\cdot\frac{(-1)\cdot \vert \alpha_{n}-\alpha _{n-1} \vert }{a(1-\rho)} \bigl( \Vert x_{n-1} \Vert + \Vert T_{n-1}x_{n-1} \Vert \bigr) \\ &\qquad{}+M_{1}\bigl( \vert t_{n}-t_{n-1} \vert + \vert \alpha _{n}-\alpha_{n-1} \vert \bigr)+ \bigl\Vert e(x_{n}) \bigr\Vert + \bigl\Vert e(x_{n-1}) \bigr\Vert , \end{aligned}$$

where $M_{1}:=\sup_{n\in\mathbb{N}} \{ \Vert h(x_{n-1}) \Vert+ \Vert T_{n-1}x_{n-1} \Vert,\frac{ \Vert x_{n-1} \Vert+ \Vert T_{n-1}x_{n-1} \Vert}{a} \}$ is well defined since $\{x_{n}\}$, $\{h(x_{n})\}$ and $\{T_{n}x_{n}\}$ are bounded.

By taking $\gamma_{n}=t_{n}(1-\rho)$, $\delta_{n}=\frac{(-1)\cdot|\alpha_{n}-\alpha_{n-1}|}{a(1-\rho)}( \Vert x_{n-1} \Vert+ \Vert T_{n-1}x_{n-1} \Vert)$ and $\beta_{n}=M_{1}(|t_{n}-t_{n-1}|+|\alpha_{n}-\alpha_{n-1}|)+ \Vert e(x_{n}) \Vert+ \Vert e(x_{n-1}) \Vert$ in (19), we get

20 $$ \Vert x_{n+1}-x_{n} \Vert \rightarrow0, \quad\mbox{as } n\rightarrow\infty $$

according to Lemma 2.7 and (i)–(iii) in Theorem 3.1.

Since $\{x_{n}\}$ is bounded, there exists a subsequence $\{x_{n_{j}}\}$ such that $x_{n_{j}}\rightharpoonup z$ as $j\rightarrow\infty$. In the sequel, we shall verify that $z\in S$. To this end, assume that $\alpha _{n_{j}}\rightarrow \alpha$ ($j\rightarrow\infty$), and set $T:=\operatorname{prox}_{\alpha g}(I-\alpha\nabla f)$. We compute

21 $$\begin{aligned} & \bigl\Vert x_{n_{j}}-\operatorname{prox}_{\alpha g}(I-\alpha \nabla f)x_{n_{j}} \bigr\Vert \\ &\quad \leq \Vert x_{n_{j}}-x_{n_{j}+1} \Vert + \bigl\Vert x_{n_{j}+1}-\operatorname{prox}_{\alpha g}(I-\alpha\nabla f)x_{n_{j}} \bigr\Vert \\ &\quad = \Vert x_{n_{j}}-x_{n_{j}+1} \Vert + \bigl\Vert t_{n_{j}}h(x_{n_{j}})+(1-t_{n_{j}})T_{n_{j}}x_{n_{j}}+e(x_{n_{j}}) -Tx_{n_{j}} \bigr\Vert \\ &\quad \leq \Vert x_{n_{j}}-x_{n_{j}+1} \Vert +t_{n_{j}} \bigl\Vert h(x_{n_{j}})-Tx_{n_{j}} \bigr\Vert +(1-t_{n_{j}}) \Vert T_{n_{j}}x_{n_{j}}-Tx_{n_{j}} \Vert \\ &\qquad{}+ \bigl\Vert e(x_{n_{j}}) \bigr\Vert . \end{aligned}$$

By using Lemma 2.3, we get

22 $$\begin{aligned} &\begin{aligned} &\Vert T_{n_{j}}x_{n_{j}}-Tx_{n_{j}} \Vert \\ &\quad = \bigl\Vert \operatorname{prox}_{\alpha_{n_{j}}g}(I-\alpha _{n_{j}} \nabla f)x_{n_{j}}-\operatorname{prox}_{\alpha g}(I-\alpha \nabla f)x_{n_{j}} \bigr\Vert \end{aligned} \\ &\quad = \biggl\Vert \operatorname{prox}_{\alpha g}\biggl[\frac{\alpha}{\alpha _{n_{j}}}(I- \alpha_{n_{j}}\nabla f)x_{n_{j}}+\biggl(1-\frac{\alpha}{\alpha _{n_{j}}}\biggr) \operatorname{prox}_{\alpha_{n_{j}g}}(I-\alpha_{n_{j}}\nabla f)x_{n_{j}}\biggr] \\ &\qquad{}-\operatorname{prox}_{\alpha g}(I-\alpha\nabla f)x_{n_{j}} \biggr\Vert \\ &\quad \leq\biggl\Vert \frac{\alpha}{\alpha_{n_{j}}}(I-\alpha_{n_{j}}\nabla f)x_{n_{j}} + \biggl(1-\frac{\alpha}{\alpha_{n_{j}}}\biggr)\operatorname{prox}_{\alpha _{n_{j}g}}(I- \alpha_{n_{j}}\nabla f)x_{n_{j}} \\ &\qquad{}-(I-\alpha\nabla f)x_{n_{j}} \biggr\Vert \\ &\quad = \biggl\Vert \biggl(\frac{\alpha}{\alpha_{n_{j}}}-1\biggr)x_{n_{j}} +\biggl(1- \frac{\alpha}{\alpha_{n_{j}}}\biggr)\operatorname{prox}_{\alpha _{n_{j}g}}(I- \alpha_{n_{j}}\nabla f)x_{n_{j}} \biggr\Vert \\ &\quad = \biggl\vert 1-\frac{\alpha}{\alpha_{n_{j}}} \biggr\vert \Vert x_{n_{j}}-T_{n_{j}}x_{n_{j}} \Vert \\ &\quad \leq \biggl\vert 1-\frac{\alpha}{\alpha_{n_{j}}} \biggr\vert \cdot\bigl( \Vert x_{n_{j}} \Vert + \Vert T_{n_{j}}x_{n_{j}} \Vert \bigr). \end{aligned}$$

Thus, we have

23 $$ \Vert T_{n_{j}}x_{n_{j}}-Tx_{n_{j}} \Vert \rightarrow0 \quad\mbox{as } j\rightarrow\infty $$

in view of that $\{ \Vert x_{n_{j}} \Vert\}$, $\{ \Vert T_{n_{j}}x_{n_{j}} \Vert\}$ are bounded for *j*, and $\lim_{j\rightarrow\infty}\alpha_{n_{j}}=\alpha$.

We combine (20), (21) and (23) to have

24 $$ \bigl\Vert x_{n_{j}}-\operatorname{prox}_{\alpha g}(I- \alpha\nabla f)x_{n_{j}} \bigr\Vert \rightarrow0\quad\mbox{as } j \rightarrow\infty, $$

which implies that $z\in S$ owing to Lemma 2.6, and hence $\omega_{w}(x_{n})\subset S$.

Finally, we prove that

$$\lim_{n\rightarrow\infty} \bigl\Vert x_{n}-x^{*} \bigr\Vert =0. $$

We have, by utilizing Lemma 2.4,

25 $$\begin{aligned} & \bigl\Vert x_{n+1}-x^{*} \bigr\Vert ^{2} \\ &\quad = \bigl\Vert t_{n}h(x_{n})+(1-t_{n})T_{n}x_{n}+e(x_{n})-x^{*} \bigr\Vert ^{2} \\ &\quad = \bigl\Vert t_{n}\bigl(h(x_{n})-x^{*} \bigr)+(1-t_{n}) \bigl(T_{n}x_{n}-x^{*} \bigr)+e(x_{n}) \bigr\Vert ^{2} \\ &\quad \leq \bigl\Vert t_{n}\bigl(h(x_{n})-x^{*} \bigr)+(1-t_{n}) \bigl(T_{n}x_{n}-x^{*} \bigr) \bigr\Vert ^{2}+2\bigl\langle x_{n+1}-x^{*},e(x_{n}) \bigr\rangle \\ &\quad \leq \bigl\Vert t_{n}\bigl(h(x_{n})-x^{*} \bigr)+(1-t_{n}) \bigl(T_{n}x_{n}-x^{*} \bigr) \bigr\Vert ^{2}+2 \bigl\Vert x_{n+1}-x^{*} \bigr\Vert \bigl\Vert e(x_{n}) \bigr\Vert \\ &\quad = \bigl\Vert t_{n}\bigl(h(x_{n})-h\bigl(x^{*} \bigr)\bigr)+(1-t_{n}) \bigl(T_{n}x_{n}-x^{*} \bigr)+t_{n}\bigl(h\bigl(x^{*}\bigr)-x^{*}\bigr) \bigr\Vert ^{2} \\ &\qquad{}+2 \bigl\Vert x_{n+1}-x^{*} \bigr\Vert \bigl\Vert e(x_{n}) \bigr\Vert \\ &\quad \leq \bigl\Vert t_{n}\bigl(h(x_{n})-h \bigl(x^{*}\bigr)\bigr)+(1-t_{n}) \bigl(T_{n}x_{n}-x^{*} \bigr) \bigr\Vert ^{2} \\ &\qquad{}+2\bigl\langle t_{n}\bigl(h\bigl(x^{*} \bigr)-x^{*}\bigr),x_{n+1}-e(x_{n})-x^{*} \bigr\rangle +2 \bigl\Vert x_{n+1}-x^{*} \bigr\Vert \bigl\Vert e(x_{n}) \bigr\Vert \\ &\quad \leq t_{n} \bigl\Vert h(x_{n})-h\bigl(x^{*} \bigr) \bigr\Vert ^{2}+(1-t_{n}) \bigl\Vert T_{n}x_{n}-x^{*} \bigr\Vert ^{2} \\ &\qquad{}+2t_{n}\bigl\langle h\bigl(x^{*}\bigr)-x^{*},x_{n+1}-e(x_{n})-x^{*} \bigr\rangle +2 \bigl\Vert x_{n+1}-x^{*} \bigr\Vert \bigl\Vert e(x_{n}) \bigr\Vert \\ &\quad \leq t_{n}\rho^{2} \bigl\Vert x_{n}-x^{*} \bigr\Vert ^{2}+(1-t_{n}) \bigl\Vert x_{n}-x^{*} \bigr\Vert ^{2} \\ &\qquad{}+2t_{n}\bigl\langle h\bigl(x^{*}\bigr)-x^{*},x_{n+1}-e(x_{n})-x^{*} \bigr\rangle +2 \bigl\Vert x_{n+1}-x^{*} \bigr\Vert \bigl\Vert e(x_{n}) \bigr\Vert \\ &\quad =\bigl(1-t_{n}\bigl(1-\rho^{2}\bigr)\bigr) \bigl\Vert x_{n}-x^{*} \bigr\Vert ^{2}+2t_{n} \bigl\langle h\bigl(x^{*}\bigr)-x^{*},x_{n+1}-x^{*} \bigr\rangle \\ &\qquad{}-2t_{n}\bigl\langle h\bigl(x^{*}\bigr)-x^{*},e(x_{n}) \bigr\rangle +2 \bigl\Vert x_{n+1}-x^{*} \bigr\Vert \bigl\Vert e(x_{n}) \bigr\Vert \\ &\quad \leq\bigl(1-t_{n}\bigl(1-\rho^{2}\bigr)\bigr) \bigl\Vert x_{n}-x^{*} \bigr\Vert ^{2}+2t_{n} \bigl\langle h\bigl(x^{*}\bigr)-x^{*},x_{n+1}-x^{*} \bigr\rangle \\ &\qquad{}+2t_{n} \bigl\Vert h\bigl(x^{*} \bigr)-x^{*} \bigr\Vert \bigl\Vert e(x_{n}) \bigr\Vert +2 \bigl\Vert x_{n+1}-x^{*} \bigr\Vert \bigl\Vert e(x_{n}) \bigr\Vert \\ &\quad =\bigl(1-t_{n}\bigl(1-\rho^{2}\bigr)\bigr) \bigl\Vert x_{n}-x^{*} \bigr\Vert ^{2}+2t_{n} \bigl\langle h\bigl(x^{*}\bigr)-x^{*},x_{n+1}-x^{*} \bigr\rangle \\ &\qquad{}+2\bigl(t_{n} \bigl\Vert h\bigl(x^{*} \bigr)-x^{*} \bigr\Vert + \bigl\Vert x_{n+1}-x^{*} \bigr\Vert \bigr) \bigl\Vert e(x_{n}) \bigr\Vert \\ &\quad \leq\bigl(1-t_{n}\bigl(1-\rho^{2}\bigr)\bigr) \bigl\Vert x_{n}-x^{*} \bigr\Vert ^{2}+2t_{n} \bigl\langle h\bigl(x^{*}\bigr)-x^{*},x_{n+1}-x^{*} \bigr\rangle \\ &\qquad{}+M_{2} \bigl\Vert e(x_{n}) \bigr\Vert , \end{aligned}$$

where $M_{2}=\sup_{n\in\mathbb{N}}\{2(t_{n} \Vert h(x^{*})-x^{*} \Vert + \Vert x_{n+1}-x^{*} \Vert)\}<\infty$ in view of $t_{n}\in[0,1]$ and $\{x_{n}\}$ being bounded.

In order to apply Lemma 2.7 to (25), we need to prove

26 $$ \limsup_{n\rightarrow\infty}\bigl\langle h\bigl(x^{*} \bigr)-x^{*},x_{n}-x^{*}\bigr\rangle \leq0. $$

Select a suitable subsequence $\{x_{n_{i}}\}$ from $\{x_{n}\}$ such that

27 $$ \limsup_{n\rightarrow\infty}\bigl\langle h \bigl(x^{*}\bigr)-x^{*},x_{n}-x^{*}\bigr\rangle =\lim_{i\rightarrow\infty}\bigl\langle h\bigl(x^{*} \bigr)-x^{*},x_{n_{i}}-x^{*}\bigr\rangle . $$

Since $\{x_{n_{i}}\}$ is bounded, it has a weakly convergent subsequence. Without loss of generality, we denote the weakly convergent subsequence by $\{x_{n_{i}}\}$ and assume that $x_{n_{i}}\rightharpoonup\hat{z}$. Then $\hat{z}\in S$, and

28 $$\begin{aligned} &\limsup_{n\rightarrow\infty}\bigl\langle h\bigl(x^{*} \bigr)-x^{*},x_{n}-x^{*}\bigr\rangle \\ &\quad =\lim_{i\rightarrow\infty}\bigl\langle h\bigl(x^{*} \bigr)-x^{*},x_{n_{i}}-x^{*}\bigr\rangle \\ &\quad =\bigl\langle h\bigl(x^{*}\bigr)-x^{*}, \hat{z}-x^{*}\bigr\rangle \\ &\quad \leq0. \end{aligned}$$

Take $\gamma_{n}=t_{n}(1-\rho^{2})$, $\delta_{n}=\frac{2}{1-\rho^{2}}\langle h(x^{*})-x^{*}, x_{n+1}-x^{*}\rangle$, $\beta_{n}=M_{2} \Vert e(x_{n}) \Vert$. Then all conditions in Lemma 2.7 are satisfied. Thus

29 $$ \bigl\Vert x_{n+1}-x^{*} \bigr\Vert ^{2}\leq(1-\gamma_{n}) \bigl\Vert x_{n}-x^{*} \bigr\Vert ^{2}+\gamma_{n}\delta_{n}+ \beta_{n}, $$

which implies that $x_{n}\rightarrow x^{*}$ as $n\rightarrow\infty$. □

With $\{x_{n}\}$ generated by (14), we obtain the following.

### Theorem 3.2

*With the conditions in Theorem *3.1 *hold*, *given* $x_{0}\in H$, *the sequence* $\{x_{n}\}$ *generated by* (14) *converges strongly to a point* $x^{*}\in S$.

### Proof

We complete the proof by translating (14) into the form of (12). Indeed, we can rewrite $x_{n+1}$ as

30 $$\begin{aligned} x_{n+1}&=t_{n}h(x_{n})+(1-t_{n}) \operatorname{prox}_{\alpha _{n}g}(I-\alpha_{n}\nabla f+e) (x_{n}) \\ &=t_{n}h(x_{n})+(1-t_{n})\operatorname{prox}_{\alpha_{n}g}(I- \alpha _{n}\nabla f) (x_{n})+\hat{e}(x_{n}), \end{aligned}$$

where

$$\hat{e}(x_{n}) =(1-t_{n})\bigl[\operatorname{prox}_{\alpha_{n}g}(I- \alpha_{n}\nabla f+e) (x_{n}) -\operatorname{prox}_{\alpha_{n}g}(I- \alpha_{n}\nabla f) (x_{n})\bigr]. $$

Obviously, $\Vert\hat{e}(x_{n}) \Vert\leq\Vert e(x_{n}) \Vert$ owing to Lemma 2.4 and $t_{n}\in[0,1]$. Thus we have $\sum_{n=0}^{\infty} \Vert\hat{e}(x_{n}) \Vert<\infty$. Since (12) was shown to converge, this immediately implies that (14) converges strongly to a solution of (1). □

If $e(x_{n})\equiv0$, $n\geq0$, the exact form of the two modified proximal gradient algorithms follows.

### Corollary 3.3

*With the conditions in Theorem *3.1 *holding*, *and given* $x_{0}\in H$, *arbitrarily*, *any sequence* $\{x_{n}\}$ *defined by*

31 $$ x_{n+1}:=t_{n}h(x_{n})+(1-t_{n}) \operatorname{prox}_{\alpha _{n}g}(I-\alpha_{n}\nabla f) (x_{n}), \quad n\geq0, $$

*converges strongly to a point* $x^{*}\in S$.

We also get the following result of [12, Theorem 3.2] with $T=\operatorname{prox}_{\alpha g}(I-\alpha\nabla f)$ and $\alpha _{n}\equiv\alpha$.

### Corollary 3.4

*With the conditions in Theorem *3.1 *hold*, *given* $x_{0}\in H$, *any sequence* $\{x_{n}\}$ *defined by*

32 $$ x_{n+1}:=t_{n}h(x_{n})+(1-t_{n}) \operatorname{prox}_{\alpha g}(I-\alpha \nabla f) (x_{n}), \quad n \geq0 $$

*converges strongly to a point* $x^{*}\in S$.

In addition, if *h* is some constant function, we have

### Corollary 3.5

*Under the conditions given in Theorem *3.1, *for any* $x_{0}\in H$, *the sequence* $\{x_{n}\}$ *defined by*

33 $$ x_{n+1}:=t_{n}u+(1-t_{n}) \operatorname{prox}_{\alpha_{n}g}(I-\alpha _{n}\nabla f) (x_{n}), \quad n\geq0 $$

*converges strongly to a point* $x^{*}\in S$, *where* *u* *is a point in* *H*.

## Bounded perturbation resilience

The superiorization method can solve a broad class of nonlinear constrained optimal problems, which works by using the bounded perturbation resilience (BPR) of an original algorithm in order to steer the iterates of the algorithm towards to lower values of the objective function. In this paper, we investigate the BPR of the modified proximal gradient algorithm. The superiorization version of this scheme will be presented in the sequel paper.

Given a problem Φ. Assume that we have a basic algorithm operator $A:H\rightarrow H$, where *H* is a real Hilbert space. Then we have the following definition, which was originally given with a finite-dimensional Euclidean space [13].

### Definition 4.1

([14], Bounded perturbation resilience)

An algorithmic operator *A* is said to be bounded perturbation resilient if the following condition holds: if the sequence $\{x_{n}\}$, generated by $x_{n+1}=Ax_{n}$ with $x_{0}\in H$, converges to a solution of Φ, then any sequence $\{y_{n}\}$ generated by $y_{n+1}=A(y_{n}+\beta_{n}v_{n})$ with any $y_{0}\in H$, also converges to a solution of Φ, where the vector sequence $\{v_{n}\}_{n=0}^{\infty}$ is bounded, and the scalars $\{\beta_{n}\} _{n=0}^{\infty}$ are such that $\beta_{n}\geq0$ for all $n\geq0$, and $\sum_{n=0}^{\infty}\beta_{n}<\infty$.

If we treat the modified proximal gradient algorithm (31) as the basic algorithm A, the bounded perturbation of it is a sequence $\{x_{n}\}$ generated by

34 $$ x_{n+1}=t_{n}h(x_{n}+ \beta_{n}v_{n})+(1-t_{n})\operatorname {prox}_{\alpha_{n}g}(I-\alpha_{n}\nabla f) (x_{n}+ \beta_{n}v_{n}). $$

We have the following result.

### Theorem 4.2

*Let* *H* *be a real Hilbert space*. *Let* $h:H\rightarrow H$ *be a* *ρ*-*contractive operator*, $\rho\in(0,1)$ *and* $f,g\in\Gamma_{0}(H)$. *Assume the solution set* *S* *of* (1) *is nonempty*. *Assume*, *in addition*, *that* *f* *is differentiable*, ∇*f* *is* *L*-*Lipschitz continuous on* *H*. $\{\beta_{n}\}$, $\{v_{n}\}$ *satisfy the conditions in Definition *4.1, $\{t_{n}\}$ *and* $\{\alpha_{n}\}$ *satisfy the conditions in Theorem *3.1, *respectively*. *Then any sequence* $\{x_{n}\}$ *generated by* (34) *converges strongly to a point* $x^{*}$ *in* *S*.

*Thus*, *the modified proximal gradient algorithm is bounded perturbation resilient*.

### Proof

We rewrite (34) as

35 $$\begin{aligned} x_{n+1}&=t_{n}h(x_{n}+\beta_{n}v_{n})+(1-t_{n}) \operatorname {prox}_{\alpha_{n}g} (I-\alpha_{n}\nabla f) (x_{n}+\beta_{n}v_{n}) \\ &=t_{n}h(x_{n})+(1-t_{n})\operatorname{prox}_{\alpha_{n}g}(I- \alpha _{n}\nabla f) (x_{n})+\tilde{e}(x_{n}), \end{aligned}$$

where

$$\begin{aligned} \widetilde{e}(x_{n})&=t_{n}\bigl[h(x_{n}+ \beta_{n}v_{n})-h(x_{n})\bigr] \\ &\quad{}+(1-t_{n})\bigl[\operatorname{prox}_{\alpha_{n}g}(I- \alpha_{n}\nabla f) (x_{n}+\beta_{n}v_{n})) -\operatorname{prox}_{\alpha_{n}g}(I-\alpha_{n}\nabla f) (x_{n})\bigr]. \end{aligned}$$

In view of Lemma 2.4 and the assumptions as regards *h* and *f*, we have

36 $$\begin{aligned} & \bigl\Vert \widetilde{e}(x_{n}) \bigr\Vert \\ &\quad \leq t_{n}\Vert \beta_{n}v_{n} \Vert +(1-t_{n}) \bigl\Vert \beta_{n}v_{n} + \alpha_{n}\bigl[\nabla f(x_{n})-\nabla f(x_{n}+ \beta_{n}v_{n})\bigr] \bigr\Vert \\ &\quad \leq t_{n} \Vert \beta_{n}v_{n} \Vert +(1-t_{n}) (1+\alpha_{n} L) \Vert \beta_{n}v_{n} \Vert \\ &\quad =\bigl[1+(1-t_{n})\alpha_{n}L\bigr] \Vert \beta_{n}v_{n} \Vert , \end{aligned}$$

which implies that $\sum_{n=0}^{\infty} \Vert\widetilde{e}(x_{n}) \Vert <\infty$ owing to the conditions imposed on $t_{n}$, $\alpha_{n}$, $\beta_{n}$ and $v_{n}$. We then deduce the conclusion from Theorem 3.1. □

## An application and the numerical experiment

In this section, we apply Theorem 4.2 to linear inverse problem and show the numerical experiment.

### Linear inverse problem

Let *H* be a real Hilbert space and $A: H\rightarrow H$ a bounded linear operator. Given $b\in H$. We consider the following linear inverse problem:

37 $$ Ax=b+w, \quad x\in H, $$

which is used to estimate an unknown signal *x* from the noise measurement *b* in finite-dimensional space. *w* is an unknown noise vector. This problem can be solved via the regularized least-squares problem:

38 $$ \min_{x\in H} \biggl\{ \frac{1}{2} \Vert Ax-b \Vert ^{2}+\gamma \Vert x \Vert \biggr\} , $$

where $\gamma>0$ is a regularization parameter.

By applying algorithm (34) to (38), we obtain the following.

#### Theorem 5.1

*Let* $h: H\rightarrow H$ *be a* *ρ*-*contractive operator with* $\rho\in(0,1)$. *Assume* $A\neq0$ *and the solution set* *S* *of* (38) *is nonempty*. *Assume*, *in addition*, $\{v_{n}\}$ *is a bounded sequence in* *H*, $\{\beta_{n}\}\subset(0,+\infty)$ *such that* $\sum_{n=0}^{\infty}\beta _{n}<\infty$. *Given* $x_{0}\in H$, *we define* $\{x_{n}\}$ *by the iterative scheme*

39 $$ x_{n+1}=t_{n}h(x_{n}+ \beta_{n}v_{n})+(1-t_{n})\operatorname {prox}_{\alpha_{n}\gamma \Vert \cdot \Vert }\bigl(I-\alpha_{n}A^{*} \bigl(A(x_{n}+\beta_{n}v_{n})-b\bigr)\bigr) $$

($A^{*}$ *is the adjoint of A*), *where*

(i) $0< a\leq\alpha_{n}<\frac{2}{ \Vert A \Vert^{2}}$, $\sum _{n=0}^{\infty} |\alpha_{n+1}-\alpha_{n}|<\infty$;
(ii) $\{t_{n}\}\subset(0,1)$, $\lim_{n\rightarrow\infty}t_{n}=0$;
(iii) $\sum_{n=0}^{\infty}t_{n}=\infty$, $\sum_{n=0}^{\infty }|t_{n+1}-t_{n}|<\infty$.

*Then* $\{x_{n}\}$ *converges strongly to a point* $x^{*}\in S$, *where* $x^{*}$ *is the unique solution of the following variational inequality problem*:

$$ \bigl\langle (I-h)x^{*},x-x^{*}\bigr\rangle ,\quad\forall x\in S. $$

#### Proof

Take $f(x)=\frac{1}{2} \Vert Ax-b \Vert^{2}$, $g(x)=\gamma\Vert x \Vert$. It is easy to see that $f,g\in\Gamma_{0}(H)$, $\nabla f(x)=A^{*}(Ax-b)$, and

40 $$ \begin{aligned}[b] & \bigl\Vert \nabla f(x)-\nabla f(y) \bigr\Vert \\ &\quad = \bigl\Vert A^{*}(Ax-b)-A^{*}(Ay-b) \bigr\Vert = \bigl\Vert A^{*}A(x-y) \bigr\Vert \leq \Vert A \Vert ^{2} \Vert x-y \Vert . \end{aligned} $$

So ∇*f* is Lipschitz continuous with $L= \Vert A \Vert^{2}$. In addition, *g* is subdifferentiable, and its subdifferential is

41 $$ \partial \Vert \cdot \Vert (x)= \textstyle\begin{cases}\{x/ \Vert x \Vert \}, &\mbox{if } x\neq0;\\ B(0;1), &\mbox{if } x=0. \end{cases} $$

So we can apply Theorem 4.2 to obtain this result. □

### Numerical experiment

In this subsection, we apply the iterative scheme (34) to solve (38) with $H=\mathbb{R}^{J}$ to demonstrate the effectiveness of this algorithm. For finite-dimensional spaces, the least-squares problem (38) takes the form as follows:

42 $$ \min \biggl\{ \frac{1}{2} \Vert Ax-b \Vert _{2}^{2}+\gamma \Vert x \Vert _{1}: x\in \mathbb{R}^{J} \biggr\} , $$

where $A\in\mathbb{R}^{M\times J}$ is a matrix. The vector $b\in\mathbb {R}^{M}$. $f(x)=\frac{1}{2} \Vert Ax-b \Vert_{2}^{2}$ implies $\nabla f(x)=A^{T}(Ax-b)$ with $L= \Vert A^{T}A \Vert$, where $A^{T}$ represents the transpose of *A*. $\operatorname{prox}_{\alpha_{n}\gamma\Vert\cdot\Vert_{1}}(x_{n}) =(\operatorname{prox}_{\alpha_{n}\gamma|\cdot|_{1}}(x_{n}^{1}), \ldots,\operatorname{prox}_{\alpha_{n}\gamma|\cdot|_{1}}(x_{n}^{J}))^{T}$, where $\operatorname{prox}_{\alpha_{n}\gamma|\cdot|_{1}}(x_{n}^{k})=\operatorname{sgn}(x_{n}^{k}) \max\{|x_{n}^{k}|-\alpha_{n}\gamma,0\}$, $k=1,2, \ldots, J$. The bounded sequence $\{v_{n}\}$ and the summarizable nonnegative real sequence $\{\beta_{n}\}$ can be chosen as follows:

43 $$ v_{n}= \textstyle\begin{cases} -\frac{d_{n}}{ \Vert d_{n} \Vert },&\mbox{if } 0\neq d_{n}\in \partial g(x_{n}),\\ 0,& \mbox{if } 0\in\partial g(x_{n}). \end{cases} $$

$\beta_{n}=c^{n}$ for some $c\in(0,1)$.

**Figure 1:**
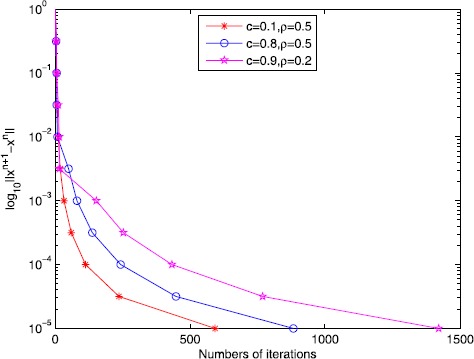
The numbers of iterations under the different error values

Throughout the experiments, $A\in\mathbb{R}^{M\times J}$ is a matrix whose entries are sampled independently from a Gaussian distribution of zero mean and unit variance. The vector $b\in\mathbb {R}^{M}$ is generated from a uniformly distribution in the interval $[-5,5]$. The regularization parameter $\gamma=0.05$. We choose $M=50$ and $J=200$. Given $t_{n}=\frac{1}{3n}$, $\alpha_{n}=\frac{n}{3\sqrt{L}(n+1)}$, we define the stopping criterion

44 $$ \mathit{Err}:= \Vert x_{n+1}-x_{n} \Vert < \varepsilon, $$

where *ε* is a given small positive constant. To see the behavior of algorithm (34), we plotted the evolutions of ‘Err’ defined by (44) with respect to the numbers of iterations in Fig. 1 for the initial point $x_{0}=(0,0,\ldots,0)^{T}\in\mathbb{R}^{200}$. The plots in Fig. 1 show that the proposed algorithm is reliable to solve (42). Besides, The iteration numbers (“Iter”), the computing time in seconds (“Time”), the error’s values (“Err”) and (“$\Vert Ax_{n}-b \Vert$”) are reported in Table 1 when the stopping criterion $\varepsilon=5\times10^{-5}$ is reached. We can see from Table 1 that the summarizable positive real sequence $\{\beta=c^{n}\}$ and the contractive constant *ρ* can have a large impact on the numerical performance. We also find that the sequence $\{x_{n}\}$ generated by algorithm (34) can get very close to the solution of the problem $Ax=b$.

**Table 1:** Numerical results with different $(c,\rho)$ and initial value $x_{0}$

(*c*,*ρ*)	$x_{0}=(0,0,\ldots,0)^{T}$			$x_{0}=(1,1,\ldots,1)^{T}$		
	Iter.	Time	$\Vert Ax_{n}-b \Vert$	Iter.	Time	$\Vert Ax_{n}-b \Vert$
(0.1,0.5)	152	0.140	0.066	199	0.140	0.074
(0.8,0.5)	440	0.608	0.220	260	0.172	0.070
(0.9,0.2)	680	0.546	0.307	773	0.484	0.353

## Conclusion

In this paper, we introduced a modified proximal gradient algorithm with perturbations in Hilbert space by making a convex combination of a proximal gradient operator and a contractive operator *h*. There exists a perturbation term in each iterative step (see (12)). We proved that the generated iterative sequence converges strongly to a solution of a non-smooth composite optimization problem. We also showed that the perturbation in computing the gradient of *f* in algorithm (14) actually can be seen as a special case of (12). Finally, as one of the main objectives of this paper, we verified that the exact modified algorithm is bounded perturbation resilient, a fact which, to some extent, extends the horizon of the recent developed superiorization methodology.
